# Definition and application of an assurance case development method (d*)

**DOI:** 10.1186/2193-1801-2-224

**Published:** 2013-05-16

**Authors:** Takuya Saruwatari, Shuichiro Yamamoto

**Affiliations:** Graduate School of Information Science, Nagoya University, Chikusa-ku, Nagoya, Japan; Strategy Office, Information and Communications Headquarters, Nagoya University, Chikusa-ku, Nagoya, Japan

**Keywords:** Assurance case, d*Framework, Dependability

## Abstract

Now, information systems are developed as open system that depend on each other. Assurance cases are expected to confirm a dependability of open systems. D*Framework is a method that can make assurance case for open system. In this paper we defined d*Framework formally. Furthermore we apply this method with case study and made discussion.

## Background

Dependability support of an information system is becoming difficult as the use of an information system is diversified and complicated. Recently, The system consisting of two or more portions is becoming common. We have to consider dependability of such a system. But this is difficult because we must consider multi systems simultaneously.

In recent years, creations of Assurance Case are increasing for consideration of management of dependability in systems development Kelly (1999) Sujan et al. (2007) Scott and Krombolz (2005) Bishop et al. (2004) Rhdes et al. (2009). In generally, GSN Kelly (1999) (Goal Structuring Notation), which can structurally arrange the purpose, has been used for creation of Assurance Case. In a system development process, creating and managing Assurance Case become possible by GSN. However, when two or more systems related each other, in GSN, it becomes difficult to treat the interaction between systems explicitly. In order to treat with this problem, d*Framework (d*) is proposed Yamamoto and Matsuno (2012). The d* has notion of actors. So that, it can treat a system that is consist of two or more portions. In that case, it is considered that each portion is actor. But, the definition of d* has not been given clearly yet. In this paper, we define the d* explicitly and create an assurance case as an example of case study using d*.

## Results and discussion

In this paper, we defined d* formally. And we defined creation process of d*. That result is shown in method chapter.

**Figure 1:**
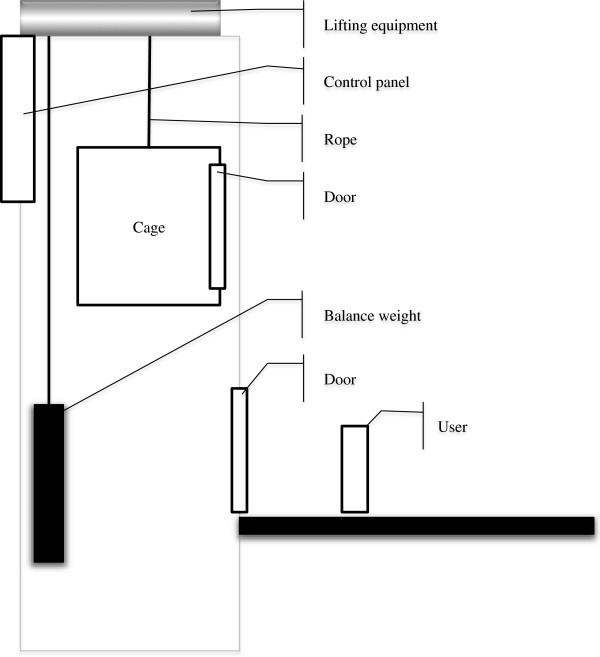
**Elevator system configuration.**

**Figure 2:**
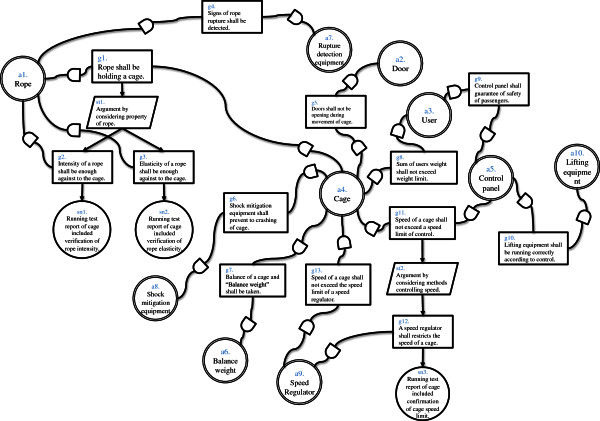
**Whole assurance case of elevator system.**

**Figure 3:**
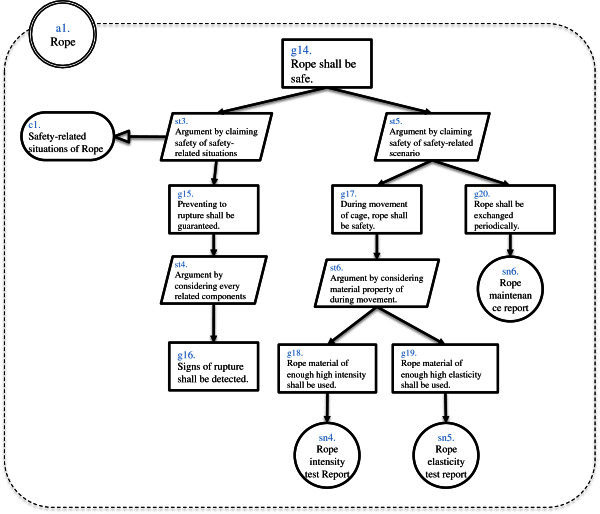
**Inner actor assurance case of rope.**

**Figure 4:**
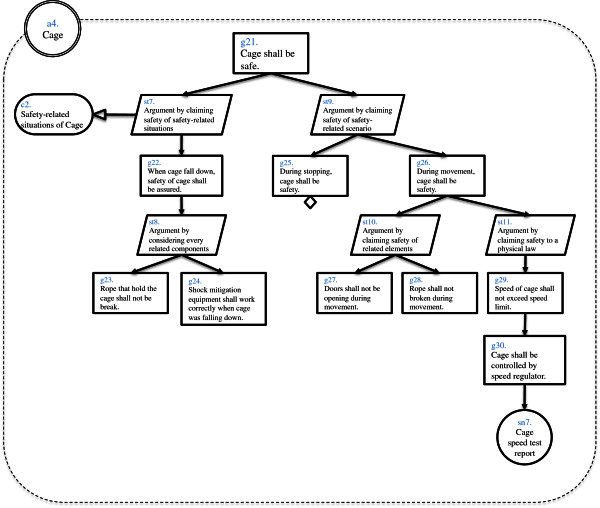
**Inner actor assurance case of cage.**

And we created Assurance Case of elevator system (Figure 1) by using d*. We created whole Assurance Case of the system and two Assurance Cases of actors. As a result we obtained dependability information as actors, goals, strategies, solutions and contexts. Numbers of element of this result are shown in Table 1. These numbers are results in a phase in the middle of Assurance Case creation. Whole Assurance Case diagram is shown in Figure 2. Assurance Case diagram of inner Actor “Rope” is shown in Figure 3 and Assurance Case of inner Actor “Cage” is shown in Figure 4. In this example “assured average” is 0.4, and “assured variance” is 0.84. “Assured average” and “assured variance” definition is shown below.

**Table 1:** **Number of elements**

	Element	Number
Actors		10
Goals	Inter Actors	13
	Inner Rope	7
	Inner Cage	10
Strategies	Inter Actors	2
	Inner Rope	4
	Inner Cage	5
Solutions	Inter Actors	3
	Inner Rope	3
	Inner Cage	1
Contexts	Inter Actors	0
	Inner Rope	1
	Inner Cage	1

We have few discussions in making Assurance Case. The discussions are shown in below.

### Effectiveness

As a case study result, it turned out that prevention of lack of consideration of dependability information is expectable. We think that expectation is derived by d* feature. The reason of that, in d*, dependability information can be elicited from between actors and can be elicited from inner actor. We think this double check of dependability information is effective for prevent lack of elicitation. In this meaning, we think that we could not elicit dependability information by using GSN. Because, when we use GSN, we did not consider notion of actor. Moreover, we think that this feature is effective to a phased test (e.g. simple substance test and joint test) that is generally carried out in system development. The accident of elevator occurred in japan before. Since the basket moved with the door opened, the accident occurred. Two subsystems were related in this accident, i.e. “Cage” and “Door” were related with this accident. If assurance case by d* was created, such an accident might have been able to been prevented, i.e. the measures to it may have been taken.

### Issues to be resolved

As a result of having applied d*, it became clear that some issues which should be solved arise.

First issue is that similar goal decomposition is performed in Inter Analysis and Inner Analysis. It may seem to have duplicate information. Here, Inter Analysis and Inner Analysis are two types of analysis in d*. This is occurred by difficulty of distinguish the aim of analysis between Inter Analysis and Inner Analysis. However, if it thinks from a viewpoint of preventing lack of analysis of dependability information, we think that it will be thought that this problem is permissible.

Second issue is that diagram of Assurance Case becomes too big and complicated. Although it is only an early stage of the construction of Assurance Case shown in this paper, it is too big and complicated diagram enough. If creation progresses after this, the diagram will become much more complicated. So, understanding of whole assurance case will be very difficult. And progression of create of the assurance case will be difficult. Given the full-fledged use, we think that development of support tools that can solve this issue in the future will be a challenge. The D-Case editor D-Case editor shown in related work is expected as a means to conquer this challenge.

### Comparison of methods

There are several methods that treat dependability information. So, we compared five methods (d*, GSN, i*Framework Yu (1997), SARM, KAOS Dardenne (1993)) at four points of view. d* and GSN are method to describe assurance case. i*Framework, SARM and KAOS are method of requirement engineering. Four points are purpose, application phase, notation and information between actors. Result of comparison is shown in Table 2.

**Table 2:** **Comparison of methods**

	d*	GSN	i*Framework	SARM	KAOS
**Purpose**	Dependability guarantee of system	Dependability guarantee of system	Requirement analysis of system	Security requirement analysis of system	Requirement analysis of system
**Application phase**	Requirement definition phase,	Requirement definition phase,	Requirement definition phase	Requirement definition phase	Requirement definition phase
	Design phase,	Design phase,			
	Making phase,	Making phase,			
	Test phase,	Test phase,			
	Operation phase	Operation phase			
**Notation**	Diagram	Diagram	Diagram	Table	Diagram
**Information between Actors**	It can define goals between Actors, and can analyze of them.	-	It can define goals, tasks, resources, soft-goals between Actors.	It can define goals, tasks, resources, soft-goals between Actors, and can analyze of them.	-

Since GSN goal decomposition procedure is used inside d*, many features have overlapped between GSN and d*. It differs at the point about description of actor that is the feature of d*. If you created assurance case by GSN, you could not separate the information of actors clearly, even if the system consists of two or more subsystems. KAOS has same feature, too. So, we described “-” in “information between actors” cell of Table 2.

The five methods of Table 2 differ in the several points. Therefore, we think that each method can be complement mutually. We have to try to consider the method that is using of several methods simultaneously.

## Conclusion

In this paper, we defined d* formally. We defined creation process of d*. And, we created assurance case of elevator system by using d*. A d* has a feature that creating assurance case with consideration of actors. It is thought that this feature is useful to creation of assurance case of the system currently divided into the subsystem like open system. Furthermore, we compared five methods by four viewpoints. Five methods are d*, GSN, i*Framework, SARM and KAOS. These are different in several points, and are in complement each other. In future, we can research the way that was combined with two or more methods. Furthermore, it is necessary to evaluate the proposed method by applying it to several system developments.

## Methods

In this paper, we defined d* formally. And we defined creation process of d*. At first, we explain about an assurance case. Then we describe our definition of d*.

### Assurance case

A structured assurance case is defined as “a documented body of evidence that provides a convincing and valid argument that a specified set of critical claims regarding a system’s properties are adequately justified for a given application in a given environment” Scott and Krombolz (2005). That is, it can be think of a model created for guarantee dependability of a system. Creation of an assurance case contains aspect of a goal graph creation of requirements engineering in that a requirement is treated. However, creation of an assurance case is not only for requirements analysis. It is used in design process, making process and operation phase.

GSN is widely used as a notation for Assurance Case. Especially, there are three features in GSN.

- Definition of Strategy for goal decomposition
- Definition of Solution to the goal of the bottom of the heap
- Definition of the additional information (Justification, Assumption, Model, Context) to goals or Strategies

These features are suitable to describe the argument of dependability. However, in the model creation by GSN, there is a problem that is difficult to express appropriately Assurance Case of the open system related to mutual. So, in this paper, we use d* which can create Assurance Case of such an open systems.

### d*framework

A d* is devised in order to create an Assurance Case that took actors into consideration explicitly Yamamoto and Matsuno (2011). In d*, actor notion is introduced, like i*Framework which is one of the goal graph notation. Namely, in d*, two type of dependability information is considered. One is dependability information that exists between actors. Another is dependability information exists inside an actor.

#### Elements of d*framework

A d* has five elements for describing assurance case. These are “Actor”, “Goal”, “Strategy”, “Solution”, and “Context”.

##### Actor

Actor is an element that constitutes a system.

##### Goal

Goal shows that a system should satisfy. It can be decomposed into sub goals and sub strategies.

##### Strategy

Strategy explains argument in order to decompose Goal. It can describe means of decomposition of goal in strategy element. It can derive sub goals and sub strategies.

##### Solution

Solution is evidence that shows that goal could be satisfied.

##### Context

Context is external information that goal and strategy needed.

#### Definition of d*framework

In this paper, we defined d*Framework formally and excluded ambiguity. That definition is shown below.

##### [Def1] d*framework graphs

d*Framework graphs DF = <A, G, St, Sn, C, Rs, Rc, Rd, Rb > are consist of 9-tuples. A : actor set, G : goal set, St : strategy set, Sn : solution set, C : context set, Rs ⊆ (G∪St) × (G∪St∪Sn) : supported by relationship set, Rc ⊆ (G∪St) × C : in context of relationship set, Rd ⊆ (A∪{*}) × G × (A∪{*}) : depend on relationship set, Rb ⊆ (G∪St∪Sn∪C) × A : belong to relationship set.

Each tuple has below meaning.

- A : Set of Actors.
- G : Set of Goals. Defined inside actor shows that actor shall satisfy it. Defined between actors show that each actor agreed it.
- St : Set of strategies.
- So : Set of Solutions (Evidences).
- C : Set of contexts.
- Rs : Set of relationships between lower element (goal, strategy, solution) and upper elements. In this relationship lower elements assure upper elements. That is, upper element is decomposed to lower elements. There is transitive law in this relationship.
- Rc : Set of relationships between context and element (goal, strategy).
- Rd : Set of relationships between actors through goal. If one actor is undefined yet, undefined actor is described by “*”. Such a relationship is called “open depend on relationship”.
- Rb : Set of relationships between element (Goal, Strategy, Solution, Context) and actor. In this relationship the element belongs to the actor. This relationship is a mapping from subset of element set to actor set.

##### [Def2] Smallest depend on relationship

This relationship is derived by “open depend on relationship” and transitive law of “supported by” relationship. When there are “open depend on relationships” (<a, b, *>, <*, c, d>) and “supported by relationship” (<b, c>), relationships (<a, b, d>, <a, c, d>) are derived as “smallest depend on relationship”. This relationship should not be “open depend on relationship”.

##### [Def3] Extended depend on relationship set

This is a set of relationship that is “depend on relationship”. When there are actor A and actor B, all “depend on relationships” between A and B are included in this set.

##### [Def4] Equivalence of d* graphs

When each elements of two d* graphs are equal, it is defined that two d* graphs are equivalence.

##### [Def5] Containment of d* graphs

When there are two d* graphs DF1 and DF2. If all elements of DF1 are containment of elements of DF2, it is defined that DF1 is containment of DF2.

##### [Def6] Direct assured goal

When goal G belongs to actor and is only assured by solutions (isn’t assured by other goals), it is defined that G is “direct assured goal (DAG)”.

##### [Def7] Assured average

Sum of DAG number of each actor is “assured number (AN)” of that actor. Average of AN of all actors of d* graph is defined as “assured average (AA)”.

“Assured average” is considered as an indicator that shows a size of obligation of each actor. If assured average is big, recruitment of new actor can be considered and redistribution of DAG.

##### [Def8] Assured variance

Variance of AN of all actors of d* graph is defined as “assured variance” of that d* graph.

“Assured variance” is considered as an indicator that shows a deviation of obligation of each actor. If assured variance is big, there is a possibility that specific Actor has assured the whole assurance case. Maybe, redistribution of DAG may be needed.

#### Creation process of d*framework

**Figure 5:**
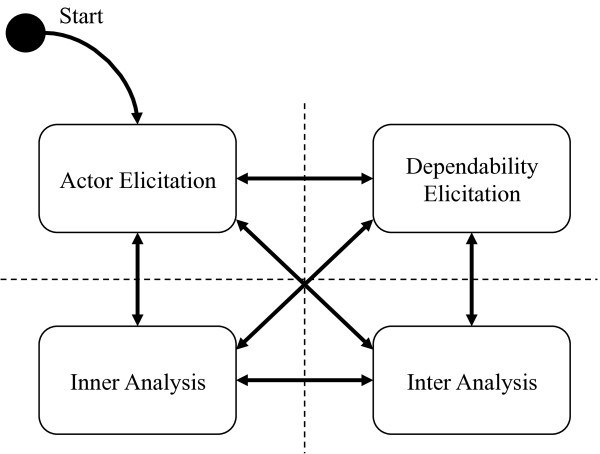
Creation process of d*.

Creation Process of d* is consist of four procedure. Each procedure is repeated mutually (Figure 5). In this creation process, creating assurance case of d* is begun at actor elicitation procedure. Explanation of four procedures is shown below.

### Actor elicitation

In this procedure, actors in a system are elicited. In the early stage of Assurance Case creation, it is elicited using the computer system configuration diagram, etc. In the stage in the middle of creation, it may be elicited as a result of an Inter Analysis procedure or an Inner Analysis procedure.

### Dependability elicitation

In this procedure, dependability information (goal) between actors is elicited.

### Inter analysis

In d*, dependability information between actors is analyzed using GSN. Analyzing to dependability information between actors is processed in this procedure. If goal that is assured single actor is elicited, Actor Elicitation procedure or Inner Analysis procedure must be processed. Namely, if the actor already existed in the assurance case, Inner Analysis procedure must be processed.

### Inner analysis

In d*, inside of actor is analyzed using GSN. If there is dependability information that is dependent from other actors to this actor, the dependability information must be satisfied in the actor. If goal depending on other actors from a target actor is elicited, it progresses to an Actor Elicitation procedure or Inter Analysis procedure.

## Related works

In recent years, many researches related to assurance case are done Kelly (1999) Sujan et al. (2007) Scott and Krombolz (2005) Bishop et al. (2004) Rhdes et al. (2009). A context notation of GSN is introduced in a research Kelly (1999). It also proposed notation for describing GSN patterns. Case study of assurance case creation is also carried out. For instance, there was a case study of the assurance case creation in the medical field Sujan et al. (2007). In these researches, GSN was used for creating assurance case. Creation of Assurance Case using GSN is not taking into consideration actor that is a subsystem as detailed unit in a system, etc. A d* that is used in this paper has actor notion. So, it can describe subsystem’s dependability information separately. And it can describe dependability information related to two or more subsystems.

In the requirements engineering, analytical methods in consideration of actors, such as i* Yu (1997) and SARM, are proposed. Moreover, also in UML (Unified Modeling Language), actor is taken into consideration by the use case diagram etc. These methods are used in requirement phase or modeling phase. It is different that d* used in all phase of system constructed.

### Dependability case editor

In order to support creation of dependability case, the D-Case editor is developed D-Case editor. In D-Case editor, an assurance case is created using extended notation of GSN. Development of the editor for supporting creation of d* is expected.
